# Frequency of the DPYD * 2A allele in the Czech population

**DOI:** 10.1186/1878-5085-5-S1-A29

**Published:** 2014-02-11

**Authors:** Monika Koudová, Martina Bittóová, Zdeňka Vlčková, Renata Alánová, Vanda Indráková, Michaela Hrabíková, Eva Lavická, Monika Hlaváčová, Karolína Perglerová, Michaela Rímska, Ilona Slepičková, Veronika Nedvědová, Lucie Pavlíčková

**Affiliations:** 1 GHC GENETICS, Ltd. - Private Medical Facility, Prague, Czech Republic

## 

The pyrimidine analogue **5-fluorouracil** (5-FU) is widely used for chemotherapy of many solid tumors, such as colorectal, breast, head/neck, ovarian and skin cancer. More than 80% of administered 5-FU is rapidly detoxified in the liver via a multistep metabolic pathway, involving **dihydropyrimidine dehydrogenase** (**DPD**) as the initial and rate-limiting enzyme. Reduced DPD activity can lead to the accumulation of active 5-FU metabolite (FdUMP), which leads to 5-FU toxicity. Individuals with low DPD activity (about 3-5% of patients) cannot effectively inactivate 5-FU and will develop severe to lethal hematological, gastrointestinal or neurological toxicities. Pharmacogenetic variation may contribute to risk of toxicity and/or altered therapeutic benefit.

Enzyme DPD has a key function for 5-FU metabolism (degradation and inactivation) and is encoded by ***DPYD* gene**. In the majority of cases, a G to A mutation in the splicing recognition sequence of intron 14 (**IVS14+1G>A**) of the DPD-encoding gene *DPYD* was shown to be responsible. The mutant allele, designated DPYD*2A, produces a nonfunctional enzyme due to skipping of exon 14. Frequence of DPYD*2A allele in caucasion population is 0,91% and in the deficite of DPD is 52%. It was recommended that screening for DPYD*2A should be routinely carried out prior to start of 5-FU therapy, and that heterozygotes should receive limited dose of 5-FU only, while homozygotes, who are at high risk to develop severe complications, should be treated with alternative therapeutic drugs.

**Figure 1:**
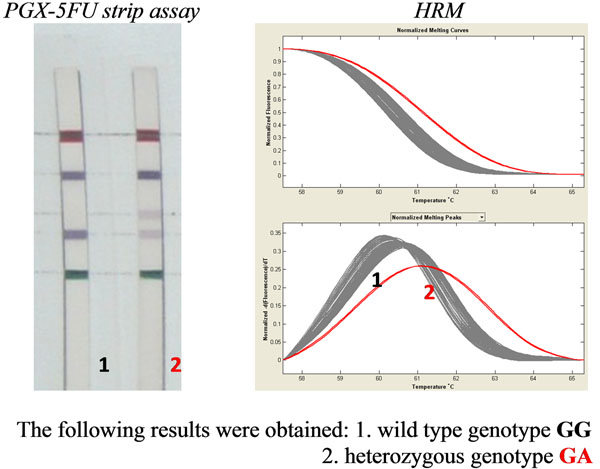
Analysis of mutation IVS14+1 G>A *DPYD* gene by methods

212 males and 210 females (the age of tested patients was 18-69 years) of the Czech population were tested. The material of DNA isolation was obtained oral mucosa by Flocked Swabs or by taking incoagulable peripheral venous blood. DNA was isolated by using QIAmp DNA Minikit or by the semi-automatic isolation DNA QuickGene 810. Mutation IVS14+1 G>A *DPYD* gene (DPYD * 2 allele) was tested by using certificated method strip assay (PGX-5FU) and by new created method High-Resolution Melting (HRM) (*Figure*1). Were compared to results of both methods with each other and assessed allele frequency in the Czech population.

DPYD * 2A allele was demonstrated in a heterozygous state in 3/422 (0.7%), in the homozygous state was not found. Czech frequency of DPYP * 2A allele 0.36% is 2.5 times lower than the reported frequency 0.91% in the European population. The results of both methods were completely identical.

New cheaper method for detection DPYD * 2A was introduced and validated. Low frequency of DPYD * 2A allele in the Czech population assumes the presence of other mutations in the *DPYD* gene and in other genes affecting the metabolism of 5-FU (eg . *TYMS*, *MTHFR*).

**Genetic test is recommended prior to cancer treatment** by 5-FU, because are suitable the lower doses in the heterozygotes and choose other chemotherapy for the homozygotes with risk of toxic reaction.

